# Oxic methane production from methylphosphonate in a large oligotrophic lake: limitation by substrate and organic carbon supply

**DOI:** 10.1128/aem.01097-23

**Published:** 2023-11-30

**Authors:** Logan M. Peoples, John E. Dore, Evan M. Bilbrey, Trista J. Vick-Majors, John R. Ranieri, Kate A. Evans, Abigail M. Ross, Shawn P. Devlin, Matthew J. Church

**Affiliations:** 1 Flathead Lake Biological Station, University of Montana, Polson, Montana, USA; 2 Department of Land Resources and Environmental Sciences, Montana State University, Bozeman, Montana, USA; 3 Department of Biological Sciences, Idaho State University, Pocatello, Idaho, USA; 4 Department of Biological Sciences, Michigan Technological University, Houghton, Michigan, USA; Georgia Institute of Technology, Atlanta, Georgia, USA

**Keywords:** methane, methylphosphonate, lake, freshwater, *Acidovorax*, phosphorus

## Abstract

While methane is typically produced under anoxic conditions, methane supersaturation in the presence of oxygen has been observed in both marine and fresh waters. The biological cleavage of methylphosphonate (MPn), which releases both phosphate and methane, is one pathway that may contribute to this paradox. Here, we explore the genomic and functional potential for oxic methane production (OMP) via MPn in Flathead Lake, a large oligotrophic freshwater lake in northwest Montana. Time series and depth profile measurements show that epilimnetic methane was persistently supersaturated despite high oxygen levels, suggesting a possible *in situ* oxic source. Metagenomic sequencing indicated that 10% of microorganisms in the lake, many of which are related to the Burkholderiales (Betaproteobacteria) and Actinomycetota, have the genomic capacity to cleave MPn. We experimentally demonstrated that these organisms produce methane stoichiometrically with MPn consumption across multiple years. However, methane was only produced at appreciable rates in the presence of MPn when a labile organic carbon source was added, suggesting that this process may be limited by both MPn and labile carbon supply. Members of the genera *Acidovorax*, *Rhodoferax*, and *Allorhizobium*, organisms which make up less than 1% of Flathead Lake communities, consistently responded to MPn addition. We demonstrate that the genomic and physiological potential for MPn use exists among diverse, resident members of Flathead Lake and could contribute to OMP in freshwater lakes when substrates are available.

**IMPORTANCE:**

Methane is an important greenhouse gas that is typically produced under anoxic conditions. We show that methane is supersaturated in a large oligotrophic lake despite the presence of oxygen. Metagenomic sequencing indicates that diverse, widespread microorganisms may contribute to the oxic production of methane through the cleavage of methylphosphonate. We experimentally demonstrate that these organisms, especially members of the genus *Acidovorax*, can produce methane through this process. However, appreciable rates of methane production only occurred when both methylphosphonate and labile sources of carbon were added, indicating that this process may be limited to specific niches and may not be completely responsible for methane concentrations in Flathead Lake. This work adds to our understanding of methane dynamics by describing the organisms and the rates at which they can produce methane through an oxic pathway in a representative oligotrophic lake.

## INTRODUCTION

Globally, freshwaters account for approximately 20% of methane (CH_4_) emissions to the atmosphere (1). The conventional understanding of biological methanogenesis is that it occurs exclusively under anoxic conditions (2, 3). However, in the upper ocean and the epilimnia of freshwater lakes where oxygen is present, concentrations of methane can be supersaturated with respect to atmospheric equilibrium (4–8). A growing number of studies using measurements of methane stable isotopic composition and physical transport modeling indicate that supply from anoxic habitats may not be responsible for these elevated concentrations (7, 9). While many mechanisms for methane production in oxic waters have been suggested, including photosynthesis, the metabolism of methylated amines, and within cryptic anoxic niches (10–13), the degree to which these pathways contribute to methane supersaturation is unknown. Our understanding of the production of CH_4_ and its contribution to atmospheric concentrations is important as methane flux from lakes is projected to increase in future climate change scenarios (14, 15).

Many lakes appear phosphorus limited (16, 17), potentially inducing the use of alternative phosphorus sources other than phosphate by microorganisms. A portion of the dissolved organic matter pool in marine and freshwater environments resides as dissolved organic phosphorus (DOP), a poorly characterized reservoir of carbon and phosphorus (18). DOP can include phosphonates, compounds characterized by a stable C-P bond that is synthesized as part of lipid headgroups, exopolysaccharides, and glycoproteins (19). These compounds are produced by some of the most abundant lineages in the global ocean, including SAR11, *Prochlorococcus*, and *Nitrosopumilus* (20–23). While phosphonates and the transcription of genes involved in their synthesis have been detected in freshwater ecosystems (10, 24–29), quantitative determinations of their types, abundances, and distributions are sparse. One type of phosphonate is methylphosphonate (MPn), the demethylation of which releases not only phosphate but also methane. Oxic methane production (OMP) via the degradation of MPn has helped explain the methane paradox in the upper ocean (30–34). Organisms in freshwater and meromictic lakes can also cleave MPn, including members of the Alphaproteobacteria, Gammaproteobacteria, and Betaproteobacteria (35–37). However, the contribution of this process to methane production in phosphorus-limited freshwater systems and the diversity of organisms catalyzing it have not been well documented.

We explored the dynamics of methane and the microbial capacity to use MPn in Flathead Lake, Montana, one of the largest (surface area ~500 km^2^, maximum depth 116 m) natural freshwater lakes in the western United States. Its short hydrologic residence time (~2.2 years) (38) and environmentally protected, largely undeveloped montane watershed make Flathead Lake oligotrophic; soluble reactive phosphorus levels are typically below detection, and N:P stoichiometric ratios are elevated (39). Experimentally, plankton growth in the lake can be limited by nitrogen, phosphorus, or light (39–42). A large fraction of the available nitrogen and phosphorus may be in the form of dissolved organic matter (39, 43). In this study, we sought to address four questions: (i) How do concentrations of methane vary over space and time in Flathead Lake? (ii) What is the taxonomic and temporal distribution of organisms that have the genomic capacity for methylphosphonate-mediated methane production? (iii) Can we show experimentally that these organisms can perform OMP? (iv) What factors may limit the magnitude of OMP *in situ*?

## RESULTS

### Methane dynamics and production in Flathead Lake

**Fig 1:**
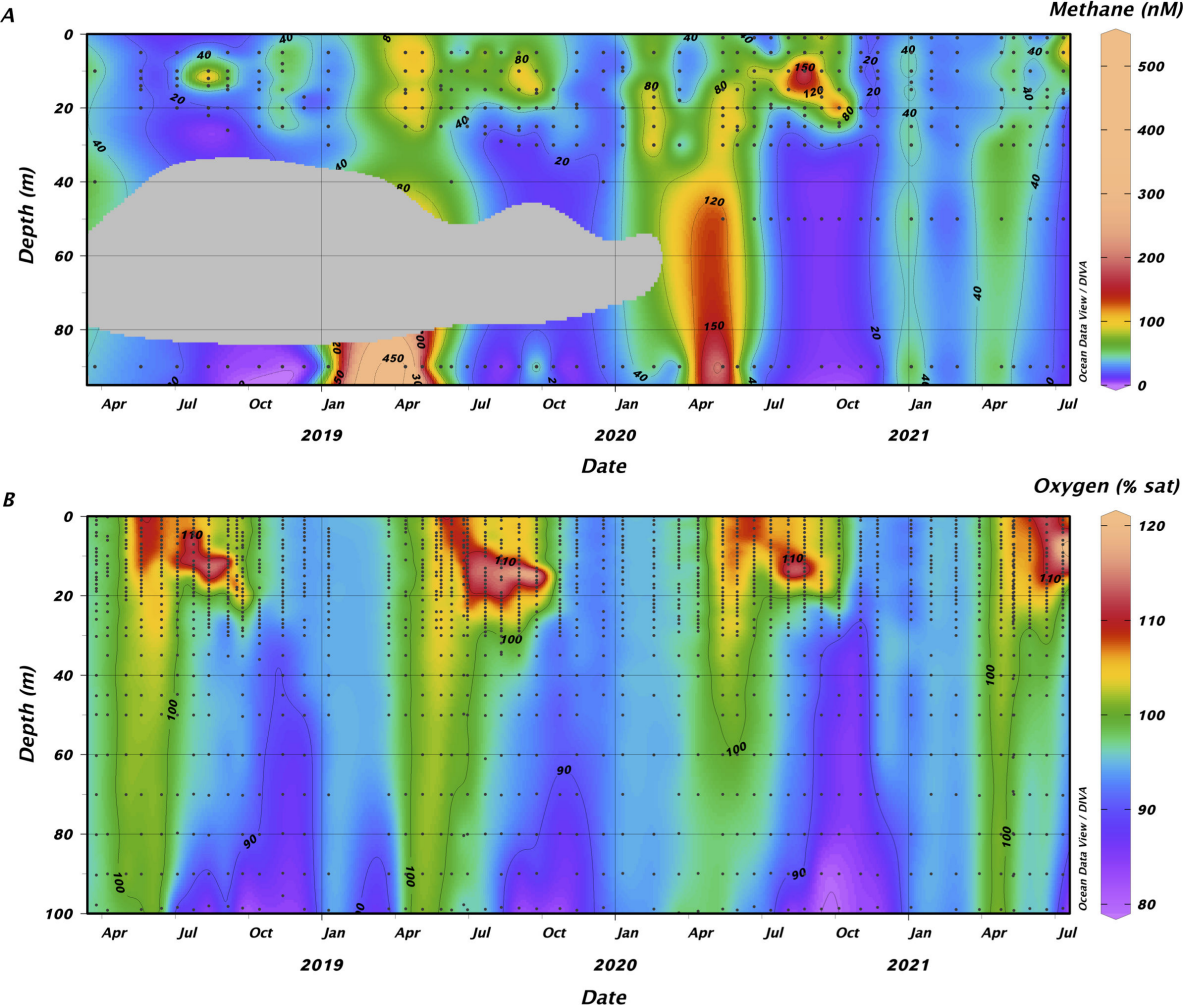
Methane is present and temporally variable, despite the consistent presence of oxygen, in Flathead Lake, Montana. Methane concentrations (**A**) and oxygen percent saturation (**B**) in Flathead Lake over a 3-year period. Black dots reflect sampling points. The gray area reflects dates and depths where no methane measurements were conducted.

To evaluate the possibility of OMP occurring in Flathead Lake, we investigated the dynamics of methane and oxygen concentrations in Flathead Lake over a 3-year period (Fig. 1). Sampling was conducted at the long-term monitoring site Midlake Deep (MLD), one of the deepest points in the lake (~113 m). The lake was persistently oxic, with oxygen percent saturation rarely falling below 80%. Methane concentrations typically ranged from 20 to 200 nM and were consistently oversaturated with respect to atmospheric levels. Methane was generally highest and evenly distributed across all depths during the spring. As the summer progressed, a subsurface methane maximum developed. Concentrations were elevated throughout the upper 10–15 m and peaked within the upper thermocline in August and September, suggesting an *in situ* source of methane within the epilimnion.

### Methane-related microbial diversity in Flathead Lake

**Fig 2:**
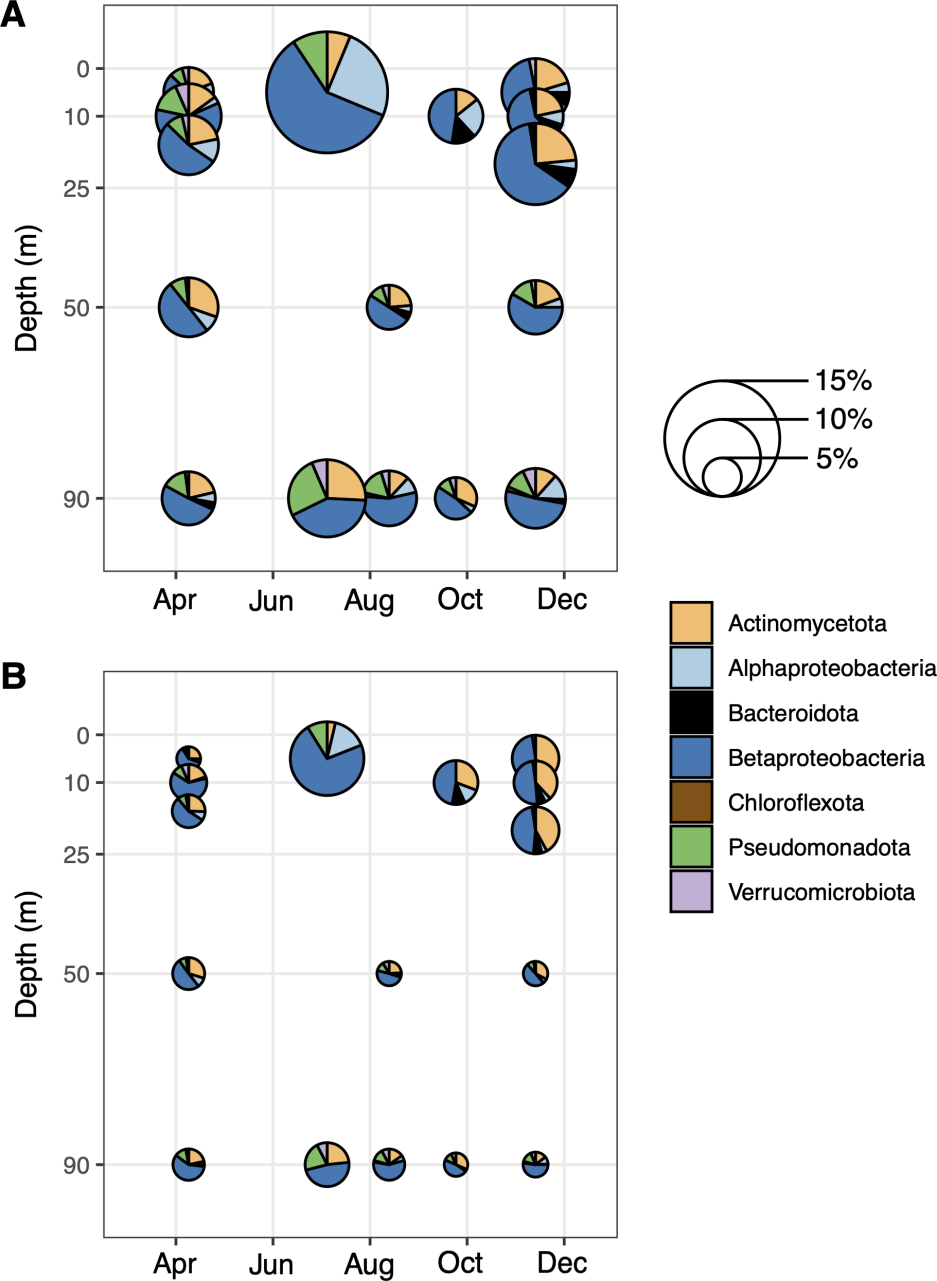
Members of the Betaproteobacteria and Actinomycetota are the most diverse and abundant *phnJ*-containing organisms in Flathead Lake (metagenomic sequencing from samples collected in 2018). (**A**) Relative abundance of species that have *phnJ* relative to the total number of unique species estimated from single-copy marker genes. (**B**) Relative abundance of microorganisms within the community which have *phnJ* relative to single-copy marker gene abundances based on read recruitment. Relative abundances at each time point are designated by circle size, while the colors represent the proportion of that relative abundance attributed to a given taxonomic group. Circle size and colors are the same in both panels.

We performed metagenomic sequencing to explore the potential for methane generation in Flathead Lake during 2018 (Table S1). The methyl coenzyme M reductase (*mcrA*) gene, a diagnostic marker for methanogenesis (44), and known methanogenic archaea were absent in our metagenomic and 16S rRNA amplicon data sets. In contrast, we found that up to 15% of the species in Flathead Lake had the capacity to cleave MPn, producing phosphate and methane, using the C-P lyase gene *phnJ* (Fig. 2). When accounting for the relative abundances of these organisms, they represented between 2% and 10% of the total overall community. The majority of the *phnJ* diversity was represented by sequences related to the Betaproteobacteria (order Burkholderiales; some databases now classify them as Gammaproteobacteria) and Actinomycetota (previously Actinobacteria or Actinobacteriota; class Ilumatobacteraceae) but also included members of the Alphaproteobacteria, Bacteroidota (previously Bacteroidetes), other Pseudomonadota (Proteobacteria), and Verrucomicrobiota. While the number of organisms with *phnJ* generally appeared higher within the epilimnion than in deeper waters, neither the percentage of species (Pearson correlation, *R* < 0.1, *P* > 0.9) or percentage of organisms (Pearson correlation, *R* < 0.1, *P* > 0.75) correlated with methane concentrations. These observations were consistent when looking at the distributions of other genes in the *phn* operon.

We screened the *in situ* metagenomes for the capacity for phosphonate production and alternative pathways of methane cycling. Members of the phylum Nitrospirota (previously Nitrospirae) and the family Rhodospirillales (Alphaproteobacteria) possessed the *pepM* gene for the first step in phosphonate biosynthesis. These genes were present in the deep waters of the lake. We also identified putative, divergent *pepM* genes related to members of the Dehalococcoideae, Ignavibacteriae, Bacteroidota, Bacillota, Woesarchaeota, Pseudomonadota (<70% similar to *Pelagibacter*), and *Polynucleobacter*. These sequences generally showed less than 70% similarity to all currently published sequences. The gene *ppd* (*aepY*), involved in the conversion of Pn-pyruvate to Pn-acetylaldehyde, was also divergent in our metagenomes, with genes often showing less than 50% similarity to sequences belonging to the Deltaproteobacteria, Alphaproteobacteria, Woesarchaeota, Nitrospirota, Verrucomicrobiota, Bacillota, and Flavobacteriaceae within the Bacteroidota. We were unable to find the *mpnS* gene, which encodes the protein for the final step of MPn biosynthesis, in any of the *in situ* lake metagenomes based on KEGG annotations. While not the focus of this study, we did see evidence that other types of phosphonates may also be important in freshwater systems; genes for the degradation of 2-aminoethylphosphonate (*phnW*, *phnX*) were found in abundant members of the Flathead Lake bacterial community, including the genus *Limnohabitans*, Chloroflexota (previously Chloroflexi), Nitrospirota, and Verrucomicrobiota. Notably, we identified a methane monooxygenase subunit A (*pmoA*) gene related to the Methylococcaceae, often used to identify methanotrophs, in April at multiple depths when methane concentrations were elevated. This was consistent with 16S rRNA gene amplicon sequencing which revealed that the methanotrophic family Methylococcaceae (<0.2% of all communities) was present at all depths in April but subsequently decreased to below detection the upper 15 m in the summer despite elevated methane concentrations (Fig. S1).

### Nutrient amendments

**Fig 3:**
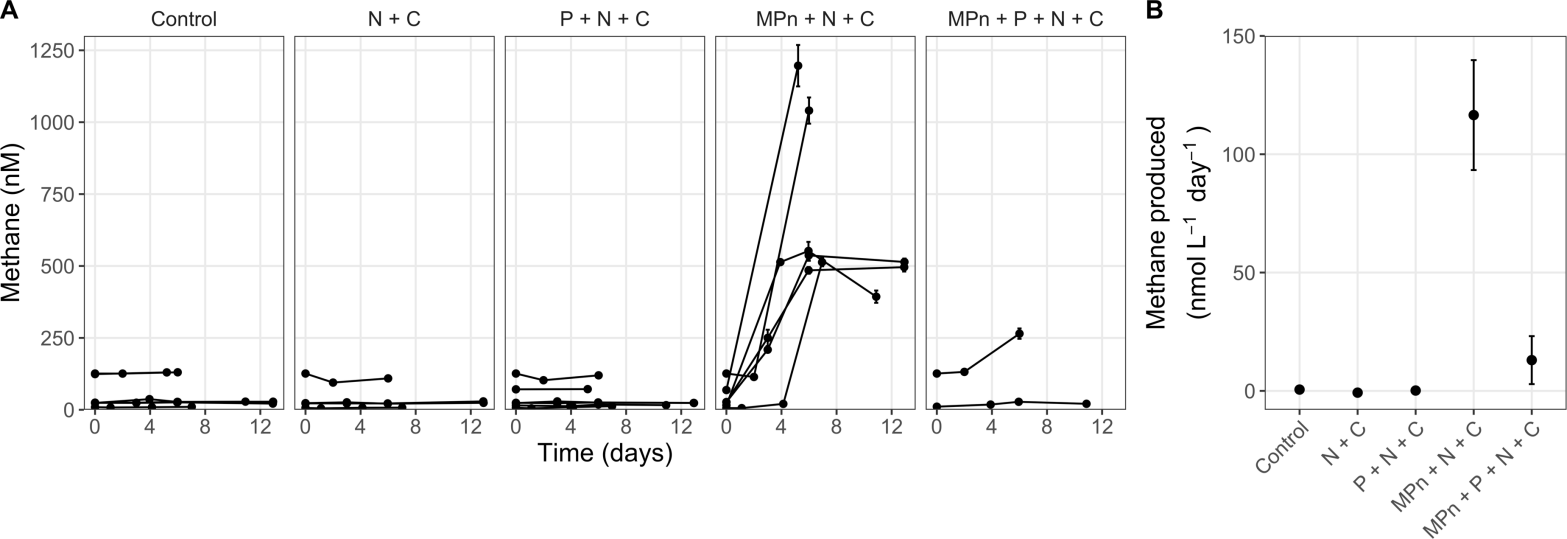
Flathead Lake summer communities (2017, 2018, and 2021) consistently produce methane when amended with methylphosphonate, nitrate, and glucose. (**A**) Time-course methane concentrations from five experiments conducted with communities from 5-m depth that were amended with different nutrients (MPn, methylphosphonate; P, phosphate; N, nitrate; C, glucose). (**B**) Average rates of methane production grouped by type of nutrient amendment. Each amendment was performed in triplicate.

Given the presence of the metabolic potential for OMP via MPn demethylation as revealed by metagenomics, we evaluated the functional capacity for this process using rate measurements of methane production under different nutrient amendments during the summers of 2017, 2018, and 2021 (Fig. 3). These experiments, using water collected from 5 m in July and August, were timed to coincide with periods when methane concentrations were elevated in the upper 10 m of the lake. Amendments included combinations of MPn, phosphate (P), nitrate (N), and glucose (C) under different light regimes (Table 1). In the unamended controls, net rates of methane production were low, averaging 0.55 ± 0.14 nmol CH_4_ L^−1^ d^−1^ (*n* = 6 experiments). Treatments amended with only N + C demonstrated net methane consumption (averaging −0.75 ± 0.69 nmol CH_4_ L^−1^ d^−1^; *n* = 4 experiments). Similarly, treatments amended with P + N + C yielded net rates of methane production that were lower than in the controls (0.15 ± 0.26 nmol CH_4_ L^−1^ d^−1^; *n* = 6 experiments). None of these treatments were significantly different than the control (Dunnett’s test, *P* > 0.05). In contrast, Flathead Lake water amended with MPn + N + C consistently produced methane after a 2- to 3-day lag time, with rates averaging 116.6 ± 23.2 nmol CH_4_ L^−1^ d^−1^ (*n* = 6 experiments; Dunnett’s test, *P* < 0.05). Methane production was correlated with the drawdown of total dissolved phosphorus (Fig. S2). In some treatments, methane concentrations plateaued at a maximum of ~0.5 µM methane despite remaining MPn. Some of these treatments also showed apparent remineralization of phosphorus over the course of the incubation, with total dissolved phosphorus (TDP) initially decreasing, but then increasing again later in the incubation period. These amendments had the lowest carbon:nitrogen:phosphorus (106:16:1 µM) concentrations added. In contrast, in treatments where carbon (250 µM) or nitrogen (30 µM) was added in excess, methane increased to final concentrations that approached the total added MPn. When samples were amended with both MPn + P, along with labile carbon and nitrogen sources, methane production rates were significantly repressed, averaging only 13.0 ± 10.1 nmol CH_4_ L^−1^ d^−1^ (*n* = 2 experiments). No differences were observed between amendments incubated in the dark, in the light, or in a dockside incubator maintaining *in situ* light and temperature fluctuations (dark, 0.58 ± 0.20 nmol CH_4_ L^−1^ d^−1^; constant or *in situ* light, 0.49 ± 0.23 nmol CH_4_ L^−1^ d^−1^; *t*-test, *P* > 0.05).

**TABLE 1:** Nutrient amendments performed using water from the epilimnion in Flathead Lake^*a*^

Start date	Unamended control	N + C	P + N	P + N + C	2P + N + C	MPn + N	MPn + N + C	2MPn + N + C	MPn + P + N + C	Conditions
2 August 2017	+	−	−	2:30:106	−	−	02:30:106	−	−	Dark, 22.5°C
2 July 2018	+	16:106	−	1:16:106	−	−	1:16:106	−	−	Dark, 22.5°C
24 July 2018	+	16:106	−	1:16:106	−	−	1:16:106	−	−	Dark and light, 22.5°C
9 August 2018	+	−	−	1:16:106	−	−	1:16:106	−	0.5:0.5:16:106	Dark, 22.5°C
20 July 2020	+	−	1:16	1:16:250	2:16:250	1:16	1:16:250	2:16:250	1:1:16:250	*In situ* light and temperature
7 July 2021	+	16:250	−	1:16:250	−	−	1:16:250	−	0.5:0.5:16:250	*In situ* light and temperature

^*a*^ All ratios indicate micromolar nutrient additions. Symbol + or a ratio indicates the amendment was performed, while symbol − means it was not performed. P, phosphate; N, nitrate; C, glucose; MPn, methylphosphonate.

**Fig 4:**
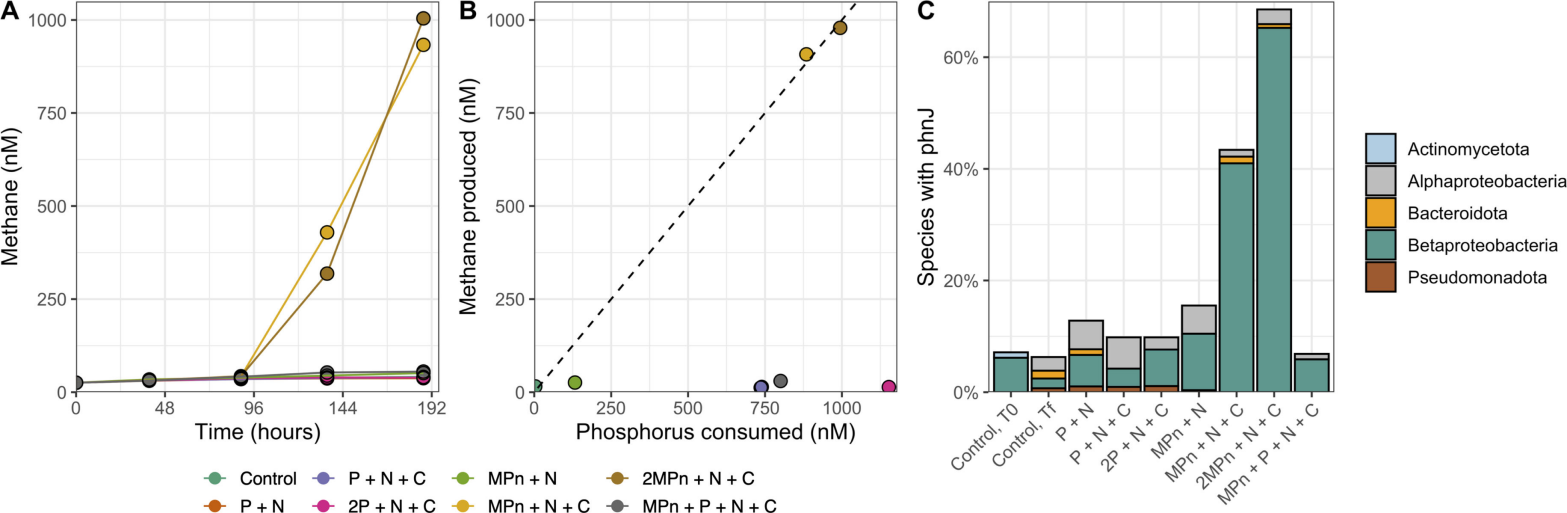
Methane is produced by members of the Betaproteobacteria (Burkholderiales) following amendment of Flathead Lake water (5-m depth) with methylphosphonate, glucose, and nitrate in July 2020. (**A**) Methane concentrations over time following amendment with different nutrients (MPn, methylphosphonate; P, phosphate; N, nitrate; C, glucose). (**B**) The total amount of methane produced relative to the amount of phosphorus consumed in each amendment. The dashed line is the 1:1 line. (**C**) The percentage of species present in each amendment which have the *phnJ* gene, colored by their taxonomic distribution. One replicate was performed for each amendment.

To evaluate the community response to MPn, we measured a more exhaustive list of properties during an amendment using lake water from 5-m depth in Summer 2020 performed under simulated *in situ* lake light and temperature conditions. This included measurements of cell abundances, chlorophyll *a* (chl *a*), nutrient concentrations, and metagenomic sequencing. Methane concentrations were highest in the upper 15 m at the time of sampling for this experiment, with *in situ* concentrations in excess of 1,000% of atmospheric equilibrium (Fig. S3). The rate of methane production without amendment was 1.51 nmol CH_4_ L^−1^ d^−1^ (Fig. 4). Methane production was not stimulated through the addition of P + N or P + N + C (1.11 and 1.32 nmol CH_4_ L^−1^ d^−1^, respectively). The addition of MPn + N also did not substantially increase methane production (2.46 nmol CH_4_ L^−1^ d^−1^). In contrast, as previously observed, the addition of MPn + N + C increased rates of net methane production to >50 nmol CH_4_ L^−1^ d^−1^, with methane produced stoichiometrically with the amount of phosphorus consumed. Consistent with the previous experiments, the addition of P alongside MPn + N + C inhibited methane production. Greater than 75% of the amended P was consumed in the MPn + N + C, P + N + C, and P + N treatments where 1 µM P was added (Fig. 4; Fig. S4). In contrast, less than ~10% of the available phosphorus and nitrate were consumed in the MPn + N treatment. TDP also remained in treatments where 2 µM phosphorus was added, likely reflecting carbon or nitrogen limitation.

### Community composition following amendment

We performed metagenomic sequencing to identify the microorganisms which responded to the Summer 2020 amendment which simulated *in situ* lake light and temperature conditions. Based on 16S rRNA gene composition, at the onset of the experiment the *in situ* lake community contained high abundances of members of the Actinomycetota (Fig. S5), including sequences similar (>99%) to those from the genera *Nanopelagicus*, *Planktophila*, and other members of the hgcI clade. The most abundant 16S rRNA gene was related to the clade SAR11, identical to that obtained from Lake Superior (45) and 99.8% similar to the isolate *Fonsibacter ubiquis* (46). Abundant sequences related to the Burkholderiales included those similar to *Limnohabitans*, *Limnobacter*, *Polynucleobacter*, *Methylopumilus*, and *Rhodoferax*. Other taxa of note included those related to the Methylacidiphilaceae within the phylum Verrucomicrobiota, the cyanobacterial genus *Cyanobium*, and the *Flavobacterium* within the Bacteroidota. These organisms are consistent with those reported from other freshwater lakes (47–50), indicating that our starting community reflected the diversity typically found in freshwater systems. At the end of the experiment, the community in the unamended control was similar to the starting sample.

Nutrient amendments strongly influenced community composition. Photosynthetic and eukaryotic taxa responded to the P + N treatment based on cell abundances, chl *a* concentrations, and predicted eukaryotic contigs (Fig. S6). Cyanobacteria belonging to the genus *Cyanobium* were present, along with Bacteroidota which are known to be associated with phytoplankton blooms [e.g., references (51–53)]. Eukaryotic 18S rRNA genes also became more prevalent in the P + N treatment, including rRNA genes deriving from diatoms Bacillariophyta (*Thalassiosira*, *Skeletonema*), the green algae Chlorophyta, and the ciliate Ciliophora. In contrast, the microbial community from the treatment amended with MPn + N remained largely similar to the control community, reflected by high abundances of *Fonsibacter*, the actinobacterial hgcI clade, and diverse members of the Burkholderiales. In treatments amended with P + N + C, we observed a strong response of members of the Bacteroidota. The most abundant sequences in this treatment were related to the Spirosomaceae and included the genus *Flectobacillus* (54), an organism which can change its cell size to avoid grazing (55–58). In addition, members of the Alphaproteobacteria, including sequences related to the genus *Caulobacter* and the groups Sphingomonadales and Rhodobacteraceae, and the phylum Actinomycetota (genera *Rhodoluna* and *Aquiluna*) all responded to the P + N + C treatment. Uncharacterized sequences within the Burkholderiales, which were distantly related (<96% similar) to *Rhodoferax* and *Acidovorax*, were also present.

In contrast to the above community responses, samples amended with MPn + N + C were dominated by members of the Burkholderiales. The most abundant 16S rRNA gene sequences belonged to the genus *Acidovorax* and were similar to *Acidovorax* sp. RAC01 (59). Sequences related to *Rhodoferax*, *Curvibacter*, *Ideonella*, and *Hydrogenophaga* were also present (60, 61). Altogether, members of the Betaproteobacteria represented more than 90% of the community following amendment with MPn + N + C. The alphaproteobacterial family Rhizobiaceae also made up 1% of these communities. Despite the presence of MPn, N, and C, the MPn + P + N + C amendment was comparable to samples amended with P + N + C, showing an enrichment of Bacteroidota, Microbacteriaceae, and Sphingomonadales.

### Methylphosphonate cycling diversity

To identify organisms which responded to our treatments that had the biosynthetic capacity for methylphosphonate use, we screened the amendments for the *phnJ* gene (Fig. 4). Approximately 7% of the species in the control had the *phnJ* gene, consistent with our analyses from 2018 (Fig. 2). These sequences were similar to those reported above, including members of the Betaproteobacteria (Burkholderiales; *Rhodoferax*, *Curvibacter*, *Limnohabitans*, and *Methylopumilus*), Actinomycetota (Actinomycetia and Ilumatobacteraceae), Alphaproteobacteria (Rhodospirilalles, Caulobacteraceae, and Sphingomonadales), and Bacteroidota. In contrast, over 40% of the species in the MPn + N + C amendments had *phnJ*, showing that MPn amendment in the presence of labile carbon and nitrogen selected for microorganisms capable of using MPn. These sequences were almost entirely related to the Burkholderiales and included the genera *Acidovorax* and *Rhodoferax*. While the *in situ* lake samples also had sequences distantly related to *Acidovorax* and *Rhodoferax*, the MPn + N + C enrichment *phnJ* were unique (sharing <90% amino acid identity), indicating distinct microorganisms in these samples.

**Fig 5:**
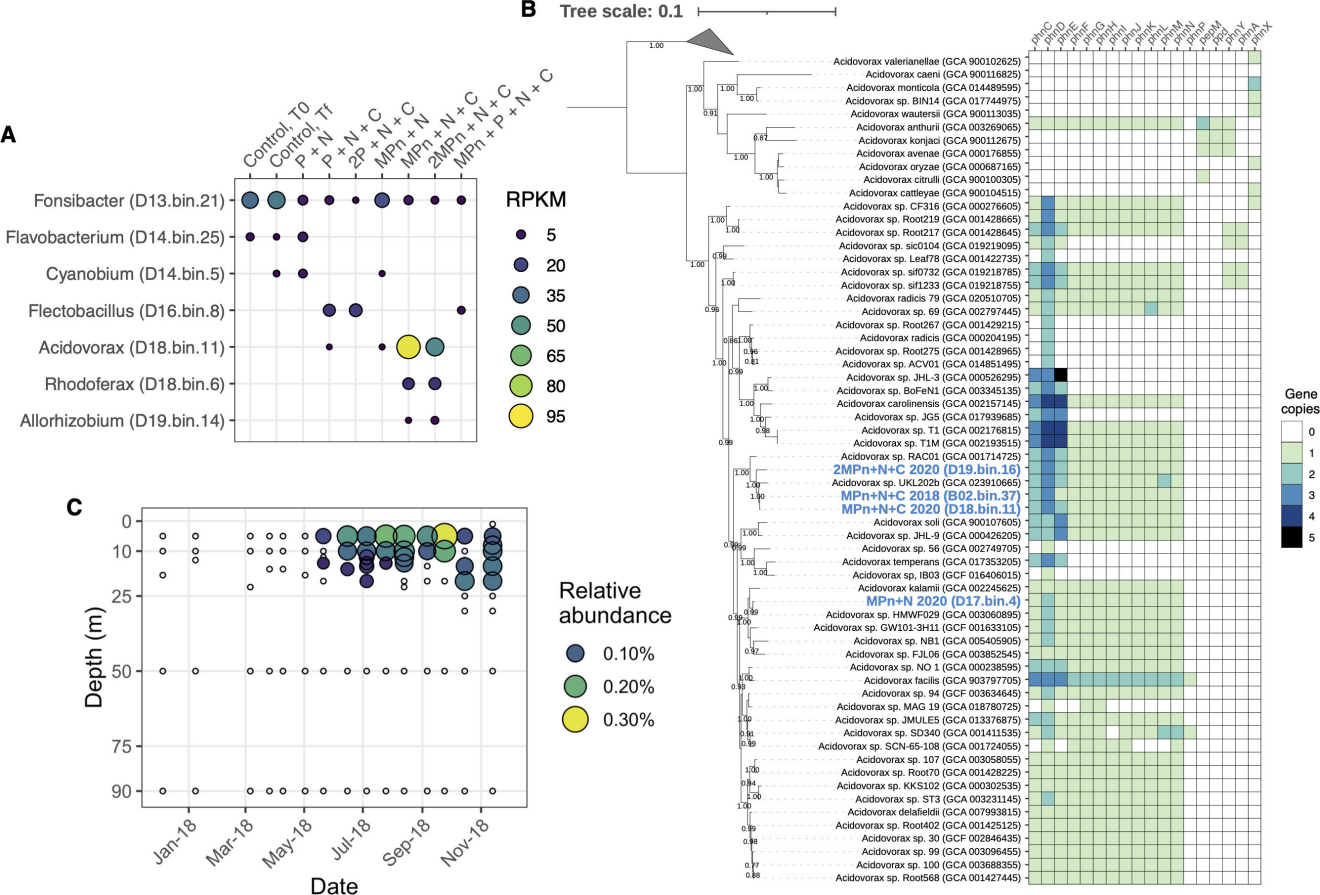
Members of the genus *Acidovorax* consistently respond to MPn addition. (**A**) Metagenomic read recruitment against seven representative MAGs obtained following nutrient amendment in July 2020. Reads per kilobase million reads (RPKM) values greater than 1 are shown. (**B**) Distribution of phosphonate cycling genes in the genus *Acidovorax*. Genomes obtained in this study are shown in blue. (**C**) Relative abundance of an *Acidovorax* 16S rRNA gene amplified sequence variant based on 16S rRNA gene amplicon sequencing of Flathead Lake which is identical to that obtained in the 2020 MPn amendment. Empty circles reflect the absence of this sequence.

To further explore the metabolic potential within each amendment, we performed genome binning (Table 2). We report metagenome-assembled genomes (MAGs) from a wide group of phyla that reflect the most abundant organisms in each treatment (Fig. 5; Fig. S7). This includes two MAGs belonging to the genus *Acidovorax* which were the most abundant genomes in the MPn + N + C and 2MPn + N + C amendments. These genomes shared 98.3% average nucleotide identity, higher than the 95% average nucleotide identity (ANI) demarcation used for identifying distinct species (62, 63). To see if the response of this organism was consistent across not only replicates but also across years, we sequenced one 2018 MPn + N + C enrichment. We discovered that the 2018 enrichment also contained an abundant *Acidovorax* MAG which shared >98.5% ANI to those from 2020. All three MAGs shared >98% ANI with strain UKL202B from Klamath Lake (64). A further *Acidovorax* genome was obtained from the MPn + N enrichment; however, this MAG shared less than 83% ANI with the other three *Acidovorax* genomes. Similarly, related yet distinct MAGs belonging to the genus *Rhodoferax* showed differential read recruitment between amendments (Fig. S7). Altogether, these findings suggest the presence of many closely related members of the Burkholderiales in Flathead Lake that may respond in distinct but reproducible ways to the availability of nutrients and organic matter.

**TABLE 2:** Metagenome-assembled genomes obtained from nutrient amendments reported in this study

Genome bin	Enrichment	Completeness (%)	Contamination (%)	Strain heterogeneity (%)	Contigs	Total length (bp)	Largest contig (bp)	N50 (bp)	GTDB-tk taxonomy
Fluviicola sp. B02.bin.61	MPn + N + C (2018)	100	0.29	0	32	4,679,305	607,597	226,288	d__Bacteria;p__Bacteroidota;c__Bacteroidia; o__Flavobacteriales;f__Crocinitomicaceae;g__Fluviicola
Acidovorax sp. D18.bin.11	MPn + N + C	99.58	4.14	28	58	4,445,087	308,013	127,350	d__Bacteria;p__Proteobacteria;c__Gammaproteobacteria; o__Burkholderiales;f__Burkholderiaceae;g__Acidovorax
Cyanobium sp. D14.bin.5	P + N	99.46	0.82	33.33	63	2,537,766	158,133	56,259	d__Bacteria;p__Cyanobacteria;c__Cyanobacteriia; o__PCC-6307;f__Cyanobiaceae;g__Cyanobium_A;
Acidovorax sp. D19.bin.16	2MPn + N + C	99.15	8.98	10.42	101	4,487,718	260,875	61,772	d__Bacteria;p__Proteobacteria;c__Gammaproteobacteria; o__Burkholderiales;f__Burkholderiaceae;g__Acidovorax
Cthoniobacterales sp. D17.bin.12	MPn + N	98.65	0	0	59	1,686,801	135,073	43,924	d__Bacteria;p__Verrucomicrobiota;c__Verrucomicrobiae; o__Chthoniobacterales;f__UBA6821;g__CAIWMF01
Rhodoferax sp. D15.bin.32	P + N + C	98.5	1.52	20	24	3,069,559	411,177	181,599	d__Bacteria;p__Proteobacteria;c__Gammaproteobacteria; o__Burkholderiales;f__Burkholderiaceae;g__Rhodoferax
Akkermansiaceae sp. D14.bin.19	P + N	98.46	0.68	0	88	3,025,235	304,644	43,437	d__Bacteria;p__Verrucomicrobiota;c__Verrucomicrobiae; o__Verrucomicrobiales;f__Akkermansiaceae;g__UBA1315
Acidovorax sp. D17.bin.4	MPn + N	97.85	2.99	26.32	139	4,692,353	111,288	52,092	d__Bacteria;p__Proteobacteria;c__Gammaproteobacteria; o__Burkholderiales;f__Burkholderiaceae;g__Acidovorax
Caulobacter sp. D15.bin.28	P + N + C	97.78	0.81	33.33	73	3,506,601	209,486	80,555	d__Bacteria;p__Proteobacteria;c__Alphaproteobacteria; o__Caulobacterales;f__Caulobacteraceae;g__Caulobacter
Prosthecobacter sp. B02.bin.14	MPn + N + C (2018)	97.62	2.04	33.33	116	5,253,014	293,079	84,038	d__Bacteria;p__Verrucomicrobiota;c__Verrucomicrobiae; o__Verrucomicrobiales;f__Verrucomicrobiaceae;g__Prosthecobacter
Verrucomicrobiales sp. B02.bin.66	MPn + N + C (2018)	96.87	0.68	0	198	4,395,267	103,091	33,241	d__Bacteria;p__Verrucomicrobiota;c__Verrucomicrobiae; o__Verrucomicrobiales;f__DEV007;g__Arctic95D-9
Limnobacter sp. B02.bin.52	MPn + N + C (2018)	96.04	0	0	21	3,279,365	559,054	305,781	d__Bacteria;p__Proteobacteria;c__Gammaproteobacteria; o__Burkholderiales;f__Burkholderiaceae;g__Limnobacter
Gammaproteobacteria sp. B02.bin.47	MPn + N + C (2018)	95.33	2.38	30.77	173	3,817,206	169,063	32,748	d__Bacteria;p__Proteobacteria;c__Gammaproteobacteria; o__Ga0077536;f__Ga0077536;g__Ga0077536
Ramlibacter sp. B02.bin.21	MPn + N + C (2018)	94.09	1.12	40	182	3,853,593	157,223	31,635	d__Bacteria;p__Proteobacteria;c__Gammaproteobacteria; o__Burkholderiales;f__Burkholderiaceae;g__Ramlibacter
Flavobacterium sp. D14.bin.25	P + N	93.82	1.32	22.22	58	2,014,991	168,531	43,385	d__Bacteria;p__Bacteroidota;c__Bacteroidia; o__Flavobacteriales;f__Flavobacteriaceae;g__Flavobacterium
Vampirovibrionales sp. B02.bin.2	MPn + N + C (2018)	93.59	1.71	0	13	2,189,344	486,615	414,704	d__Bacteria;p__Cyanobacteria;c__Vampirovibrionia; o__Vampirovibrionales;f__;g__
Fimbriimonas sp. D15.bin.35	P + N + C	93.06	0.93	0	38	2,808,834	366,894	120,327	d__Bacteria;p__Armatimonadota;c__Fimbriimonadia; o__Fimbriimonadales;f__Fimbriimonadaceae;g__Fimbriimonas
Pedosphaerales sp. B02.bin.58	MPn + N + C (2018)	92.57	2.03	40	216	3,618,279	91,808	22,155	d__Bacteria;p__Verrucomicrobiota;c__Verrucomicrobiae; o__Pedosphaerales;f__UBA9464;g__SXXZ01
Burkholderiaceae sp. B02.bin.46	MPn + N + C (2018)	91	0.36	0	218	2,647,753	57,475	14,389	d__Bacteria;p__Proteobacteria;c__Gammaproteobacteria; o__Burkholderiales;f__Burkholderiaceae;g__NBD-18
Bacteriovoracaceae sp. B02.bin.3	MPn + N + C (2018)	90.7	1.79	0	103	3,079,245	123,070	50,454	d__Bacteria;p__Bdellovibrionota;c__Bacteriovoracia; o__Bacteriovoracales;f__Bacteriovoracaceae;g__
Sphingorhabdus sp. D14.bin.29	P + N	89.86	0.72	83.33	103	2,104,408	93,469	31,795	d__Bacteria;p__Proteobacteria;c__Alphaproteobacteria; o__Sphingomonadales;f__Sphingomonadaceae;g__Sphingorhabdus_B
Aquabacterium sp. D15.bin.16	P + N + C	88.38	2.74	38.46	317	4,464,873	100,883	17,062	d__Bacteria;p__Proteobacteria;c__Gammaproteobacteria; o__Burkholderiales;f__Burkholderiaceae;g__Aquabacterium_A
Allorhizobium sp. D19.bin.14	2MPn + N + C	85.76	0.58	50	262	4,014,890	74,714	19,018	d__Bacteria;p__Proteobacteria;c__Alphaproteobacteria; o__Rhizobiales;f__Rhizobiaceae;g__Allorhizobium
Verrucomicrobiaceae sp. D15.bin.11	P + N + C	83.72	1.02	50	292	4,012,571	61,892	16,785	d__Bacteria;p__Verrucomicrobiota;c__Verrucomicrobiae; o__Verrucomicrobiales;f__Verrucomicrobiaceae;g__
Rhodoferax sp. D18.bin.6	MPn + N + C	80.54	1.32	75	143	2,736,719	148,955	24,994	d__Bacteria;p__Proteobacteria;c__Gammaproteobacteria; o__Burkholderiales;f__Burkholderiaceae;g__Rhodoferax
Aquiluna sp. D15.bin.23	P + N + C	76.36	1.46	100	76	991,789	78,207	14,987	d__Bacteria;p__Actinobacteriota;c__Actinomycetia; o__Actinomycetales;f__Microbacteriaceae;g__Aquiluna
Methylopumilus sp. D11.bin.5	Control T0	76.26	0	0	55	1,114,234	77,514	27,645	d__Bacteria;p__Proteobacteria;c__Gammaproteobacteria; o__Burkholderiales;f__Methylophilaceae;g__Methylopumilus_A
Acidovorax sp. B02.bin.37	MPn + N + C (2018)	74.86	2.08	50	244	3,202,698	67,852	16,936	d__Bacteria;p__Proteobacteria;c__Gammaproteobacteria; o__Burkholderiales;f__Burkholderiaceae;g__Acidovorax
Allorhizobium sp. B02.bin.8	MPn + N + C (2018)	74.65	1.51	37.5	368	4,128,375	69,649	12,526	d__Bacteria;p__Proteobacteria;c__Alphaproteobacteria; o__Rhizobiales;f__Rhizobiaceae;g__Allorhizobium
Methylopumilus sp. B02.bin.35	MPn + N + C (2018)	73.89	0	0	39	825,707	72,597	28,880	d__Bacteria;p__Proteobacteria;c__Gammaproteobacteria; o__Burkholderiales;f__Methylophilaceae;g__Methylopumilus
Nevskia sp. B02.bin.34	MPn + N + C (2018)	73.45	1.03	25	152	2,968,627	67,880	21,936	d__Bacteria;p__Proteobacteria;c__Gammaproteobacteria; o__Nevskiales;f__Nevskiaceae;g__Nevskia
Fonsibacter sp. D13.bin.21	Control Tf	70.08	1.2	100	64	589,623	33,776	9,058	d__Bacteria;p__Proteobacteria;c__Alphaproteobacteria; o__Pelagibacterales;f__Pelagibacteraceae;g__Fonsibacter
Rhodoferax sp. D15.bin.5	P + N + C	68.34	0.76	66.67	242	2,487,482	41,140	11,151	d__Bacteria;p__Proteobacteria;c__Gammaproteobacteria; o__Burkholderiales;f__Burkholderiaceae;g__Rhodoferax
Flectobacillus sp. D16.bin.8	2P + N + C	62.88	0.3	100	354	3,205,459	34,581	9,455	d__Bacteria;p__Bacteroidota;c__Bacteroidia; o__Cytophagales;f__Spirosomaceae;g__Flectobacillus

Given the consistent response of members of *Acidovorax* to MPn enrichment, we explored the distribution of genes involved in phosphorus use within representative members of this genus (Fig. 5). Genes involved in phosphate transport (*pstCABS*) were present in every genome. The *phn* operon (*phnFGHIJKLMNP*) was widely distributed, indicating that phosphonate use is not specific to species in Flathead Lake. Genomes of various members of the *Acidovorax* that do not possess the capacity for MPn degradation appear associated with hosts and generally fall along the lines of a recently proposed genus division (65, 66). While rare, these organisms instead have the capacity for phosphonate production, including *pepM* and *ppD*/*aepY* to convert phosphoenolpyruvate to Pn-acetylaldehyde and its subsequent degradation to either acetate or acetaldehyde using *phnYA* or *phnX*. None of the genomes had *mpnS*, the gene responsible for the final step in the production of MPn. Ultimately, we conclude that nearly every organism in the genus *Acidovorax* has some capacity for phosphonate cycling, although mechanisms of phosphonate production and MPn demethylation appear present in discrete species.

### Metagenomic and 16S rRNA amplicon abundances of methane cycling organisms

To evaluate the distributions of *phnJ*-containing *Acidovorax*, *Rhodoferax*, and *Allorhizobium* in Flathead Lake, we used metagenomic read recruitment and 16S rRNA amplicon sequencing. Metagenomic read recruitment against these MAGs showed that these organisms were rare in the *in situ* lake community, likely representing less than 1% of the total population (Fig. S8). Amplicon sequencing revealed one amplified sequence variant (ASV) identical to the dominant *Acidovorax* 16S rRNA gene sequence from our 2020 MPn + N + C amendment and shared 99.71% similarity with the 16S rRNA gene sequence from the 2018 MPn + N + C amendment. This ASV was a rare member of the Flathead Lake community, found only in the epilimnion during the summer and reached a maximum relative abundance of 0.30% (Fig. 5).

## DISCUSSION

We explored methane dynamics and the potential for MPn-mediated methane production in Flathead Lake. Despite high oxygen concentrations, methane was consistently supersaturated relative to the atmosphere. These findings indicate *in situ* methane production or a source which supplies methane to the lake. Methane concentrations ranged from 20 to 500 nM, relatively low compared to those reported in the oxygenated water column of other lakes in which OMP has been a focus [e.g., references (6, 7, 9)]. Concentrations were highest in April when the lake was fully mixed, possibly due to fluvial CH_4_ input. However, the oft-reported subsurface methane maximum also appeared in Flathead Lake as the summer progressed, suggesting an *in situ* source. Consistent with the oligotrophic nature of Flathead Lake, methane production rates in the epilimnion, based on unamended control samples, were low and averaged 0.69 ± 0.18 nmol CH_4_ L^−1^ d^−1^. These rates are significantly slower than in other lakes where OMP has been studied, including Lake Stechlin (26–236 nmol L^−1^ d^−1^) (9) and Lake Hallwil (110 nmol L^−1^ d^−1^) (67).

Given high oxygen concentrations and the apparent absence of *in situ* anaerobic methanogenesis, we sought to determine if the cleavage of MPn could be responsible for methane production. Phosphonates can comprise up to 25% of the oceanic DOP pool (18) and have been detected in lakes, although quantitative estimates are lacking and indicate they may be low (25, 26, 28). We estimate that ~10% of Flathead Lake microorganisms have the potential for MPn cleavage via C-P lyase. These abundances are similar to those in phosphorus-limited oceanic sites (23, 68), suggesting that MPn could be an important source of P in oligotrophic systems. Members of the Betaproteobacteria and Actinomycetota were the most well-represented lineages, consistent with findings in other lakes (10, 35, 37). Although the biosynthetic potential for phosphonate production appears widespread (29), genes for this process (*pepM*, *ppd*, and *mpnS*) in Flathead Lake were relatively rare. Similar findings have been reported in the ocean, where genes for the production of phosphonates are less abundant than those for its consumption (22, 23, 69). It is striking that while some of the most abundant lineages in marine systems appear capable of producing or consuming MPn, including SAR11 and *Prochlorococcus* (21, 22, 70, 71), we did not identify MPn cycling genes within Flathead Lake from members of the freshwater SAR11 *Fonsibacter* or cyanobacterial *Synechococcus*-related *Cyanobium*. Given the presence of putative phosphonate transporters in genomes of freshwater cyanobacteria (72), including those in Flathead Lake, it is possible these organisms are capable of cycling MPn using currently undescribed pathways. Regardless, it is evident that diverse microorganisms can use MPn in this system.

Following the observation of methane supersaturation and the identification of widespread genomic potential for MPn use, we experimentally demonstrated that the functional capacity for OMP exists in Flathead Lake. We found that substantial methane was only produced when both MPn and glucose were added (102.6 ± 19.4 nmol CH_4_ L^−1^ d^−1^ across all experiments). Methane was not produced following amendment with only carbon and nitrogen or MPn and nitrogen, indicating that these communities may be limited *in situ* not only by MPn substrate availability but also by organic carbon. OMP was also not observed at high rates in amendments with phosphate, even in the presence of MPn and carbon. Phosphate repression of C-P lyase has been observed in both isolates and environmental samples (35, 37, 73, 74), consistent with regulation by the Pho regulon (69). Our finding that methane production in Flathead Lake may be carbon limited is consistent with other studies which have shown that methane production is stimulated by other nutrients, including in the presence of particles and labile dissolved organic matter and through the addition of carbon, nitrogen, and even iron (31, 37, 75). Given that dissolved organic carbon in Flathead Lake is ~100 µM, similar to concentrations in the open ocean (76) where MPn is thought to contribute significantly to methane production, the type and lability of the carbon available may be important in how organisms respond to MPn. MPn cleavage may, therefore, occur at labile carbon hotspots, for example, associated with zooplankton detritus (77) or in the phycosphere, and may be a more prominent source of OMP in eutrophic lakes with excess C and N. Future work should consider the importance of the types of carbon in how and which organisms respond to MPn.

Using metagenomic sequencing, we identified the dominant microorganisms responding to MPn as members of the genera *Acidovorax* and *Rhodoferax*. Closely related strains (>98% ANI) of *Acidovorax* responded across both replicates and years and showed similarity to genomes from other lakes. These findings highlight the consistent response of *Acidovorax* to MPn, nitrate, and glucose temporally and the potential widespread distribution of this organism. Notably, members of this genus can produce methane through other aerobic pathways (78), further solidifying their role in OMP. We found that the genes for MPn use are widely distributed in this genus and did not co-occur with those for MPn synthesis, consistent with metagenomic analyses of a wide diversity of organisms from the ocean (23). In our experiments, unique strains of *Acidovorax* and *Rhodoferax* appeared to respond differently to the amendments, indicating that Flathead Lake is home to a large diversity of closely related members of the Burkholderiales that appear to differ in their nutrient and organic matter preferences. The organisms that responded to MPn amendment are rare *in situ*, likely representing <1% of the Flathead Lake community. Future transcriptomic sequencing of Flathead Lake communities will determine if these microorganisms, which show robust phosphonate utilization, are active *in situ*.

While we have shown that heterotrophic microorganisms are able to perform OMP in Flathead Lake, we conclude that net OMP is altogether very low in this system (mean 0.34 ± 0.07 nmol CH_4_ L^−1^ d^−1^ in all treatments with no added MPn; range, −2.81 to 1.59 nmol CH_4_ L^−1^ d^−1^). We are unable to rule out alternative sources of methane that could contribute to the observed supersaturation in Flathead Lake. One possibility is that methane may be produced by or associated with certain photosynthetic organisms (10, 11, 79–81). However, we did not observe strong differences in methane production in treatments in complete darkness or those with light (0.58 ± 0.20 and 0.83 ± 0.23 nmol CH_4_ L^−1^ d^−1^, respectively; *t*-test, *P* > 0.5) or in treatments where photosynthetic organisms responded. This would also not be consistent with high methane concentrations observed during April when the lake is still fully mixed. Alternatively, lateral transport from anoxic habitats (littoral, fluvial) could be a source of methane to Flathead Lake [e.g., references (82–85)]. While we provide evidence that the capacity for MPn use exists under the right conditions, namely, when both MPn and labile organic carbon are available, future work will be needed to fully understand the sources and sinks of methane in Flathead Lake.

## MATERIALS AND METHODS

Sampling was conducted at the long-term monitoring site termed Midlake Deep (47.867 N, 114.067 W) in Flathead Lake from aboard the research vessel *Jessie B*. At 113-m depth, Midlake Deep is one of the deepest points in the lake. Oxygen measurements were obtained with a Hydrolab DS5 (OTT HydroMet, Sheffield, UK) and are publicly available through the Flathead Monitoring Program (https://flbs.umt.edu/publicdata). Water samples were collected using an opaque 3-L Van Dorn water sampler or a 10-L Niskin bottle affixed to a wire and lowered via electric winch. Methane samples were fixed immediately (see below), while all other samples were stored in dark coolers during transportation back to the laboratory.

### *In situ* methane concentrations

Methane concentrations in Flathead Lake were measured approximately monthly over a 3-year period in 2018–2021. Water was placed in glass serum bottles crimp-sealed with gray chlorobutyl rubber stoppers and injection of NaOH to a final concentration of 0.1 M was used to stop biological activity. A headspace was introduced into each sample using ultra-high pure N_2_ gas followed by agitation to allow gas concentrations in the headspace to equilibrate with the liquid sample. Methane was analyzed by gas chromatography via headspace (20 mL) gas introduction (Model 8610C, SRI Instruments). Methane and oxygen concentrations were visualized and contoured using Ocean Data View (86).

### *In situ* lake metagenomic sequencing

To document the microbial diversity and potential for phosphonate use in Flathead Lake, we performed metagenomic sequencing from 16 samples collected at MLD in 2018. Depths ranged from 5 to 90 m. Approximately 1 L was serially filtered onto both a 3-µm, 25-mm GTTP polycarbonate filter (EMD Millipore, MA, USA) and a 25-mm, 0.2-µm polyethersulfone filter (SUPOR, Pall Co., NY, USA). Samples were stored at −80°C prior to DNA extraction. Genomic DNA from the <3.0 to >0.2 µm fraction was extracted using a MasterPure DNA purification kit (Lucigen, WI, USA). DNA libraries were sequenced on a Novaseq (NEB Ultra II DNA library prep kit; Novogene, Sacramento, CA), a NextSeq 2000 (Illumina DNA Prep kit; Microbial Genome Sequencing Center, Pittsburgh, PA), or a MiSeq (Nextera; Genomics Core Facility, University of Montana).

Raw reads were quality trimmed using Trimmomatic v0.39 (87) and assembled with MEGAHIT (88) using default parameters. The depth of coverage of contigs was estimated using Bowtie 2 v2.3.5.1 (89) and SAMtools v1.10 (90). Open reading frames were identified with Prodigal V2.6.3 (91). Gene annotation was performed using GhostKOALA (92). C-P lyase genes (*phnJ*; K06163) involved in MPn cleavage were classified taxonomically against the nr database using blastP (cut off 75%) (93) and through the construction of phylogenetic trees using reference sequences. Trees were created by alignment with MUSCLE (94), built using FastTree (95), and visualized using iTOL (96). We classified sequences at the phylum or class level. We estimated the percentage of species able to cleave MPn by dividing the number of *phnJ* genes by the total number of organisms estimated using four single-copy marker genes (*recA*, K03553; *gyrB*, K02470; *atpD*, K02112; *tufA*, K02358) as previously performed (23, 30, 68). We also estimated the relative abundance of the total community represented by organisms with *phnJ* by using the depth of coverage of all *phnJ* and single-copy marker genes within each metagenome. We further identified marker genes for phosphonate production (*pepM*, K01841 and K23999; *ppd*, K09459; *mpnS*, K18049), the degradation of 2-aminoethylphosphonate (*phnW*, K03430; *phnX*, K05306), anaerobic methanogenesis (*mcrA*; K00399), and methanotrophy (*pmoA*, K10944; *mmoX*, K16157).

### Nutrient amendments

To determine methane production rates under different nutrient concentrations, amendments were performed during the summers of 2017, 2018, 2020, and 2021. For all years, water was collected from MLD at a depth of 5 m, placed in 20-L polycarbonate carboys, and returned to the Flathead Lake Biological Station. Carboys were amended with MPn, P, N, and C at different final concentrations and molar ratios and incubated under specific light and temperature conditions depending on the experiment (Table 1). Carboys were subsampled into sealed borosilicate glass serum vials for subsequent incubations. Amendments from 2017 and 2018 were maintained in a laboratory incubator at ~22.5°C. While most of these amendments were maintained in the dark, one experiment (24 July 2018) was performed under both constant dark and constant light. The incubator provided photosynthetically active radiation (400–700 nm) of ~400 µmol photon m^−2^ s^−1^. To mimic *in situ* light and temperature conditions more closely, amendments in 2020 and 2021 were maintained in a dockside incubator. The incubator was plumbed to continuously supply lake water from ~2-m depth with minimal shading (at midday; ~16°C, ~70% incident photosynthetically active radiation, ~900 µmol photon m^−2^ s^−1^). A blue plastic layer made of plexiglass served to approximate the underwater spectral quality (97, 98). Amendments from 2017, 2018, and 2021 were performed using triplicate 160-mL glass serum vials with gray chlorobutyl rubber septa. All July 2020 amendments were performed with one replicate using individual 1-L glass serum bottles sealed with a blue butyl rubber septum.

Methane concentrations were followed over time to calculate rates of production. All experiments except July 2020 sacrificed whole samples at each time point. In the case of the 2020 experiment, 15 mL of sample was withdrawn and replaced with 15 mL of zero air lacking hydrocarbons. Samples were preserved with a final concentration of 0.1 M NaOH. Methane concentrations were determined as described above. Rates of methane production were calculated between equilibrated T_0_ methane concentrations and methane concentrations following 5–7 days of incubation. Because methane production showed a lag period following nutrient amendment, these rates do not necessarily reflect the maximum rates between any two time points but allow for comparison across all experiments.

For determination of nitrate + nitrite (NO_x_) and TDP, samples were filtered through MilliQ and lake water rinsed, 47-mm diameter, 0.45-µm pore size mixed cellulose ester filters, and frozen at −20°C until analysis on an Astoria A2 segmented flow analyzer (Astoria-Pacific, OR, USA). TDP was measured colorimetrically following wet chemical oxidation using an alkaline potassium persulfate digestion and treatment with heteropoly-molybdenum blue. For NO_x_ determinations, nitrate was converted to nitrite via cadmium reduction and quantified colorimetrically using Greiss chemistry.

### 2020 amendment

A more comprehensive set of measurements were performed during the amendment on 20 July 2020. These measurements are described below.

Total cell abundances and chl *a*-containing phototrophic organisms were determined using an Attune Acoustic Focusing flow cytometer (Thermo Fisher, MA, USA). Samples (2 mL) were fixed with paraformaldehyde (0.8% final concentration) and frozen at −80°C until analysis. For total cell abundances, cells were stained with the nucleic acid stain SYBR Green I for 15 minutes, excited using a 20-mW blue (488 nm) excitation laser, and detected with a 530-nm emission filter. Phototrophic cells containing chl *a* and phycoerythrin were excited using a blue (488 nm) excitation laser at 20 mW. Small phototrophic eukaryotes were detected based on chl *a* emission using a 640-nm longpass filter, while cyanobacteria containing phycoerythrin were distinguished using an R-phycoerythrin emission filter (574 nm). Concentrations of chl *a* were determined following filtration of lake water (100 mL) onto a 25-mm diameter 0.45-µm pore size mixed cellulose ester filter (Millipore). Filters were extracted at −20°C in a 90% acetone solution overnight and the fluorescence quantified using a Turner 10-AU fluorometer (Turner Designs, CA, USA), including phaeophytin correction (99).

We performed metagenomic sequencing to determine community shifts in response to the experimental amendments. Approximately 100–500 mL of water was filtered onto a 25-mm diameter, 0.2-µm polyethersulfone filter (SUPOR, Pall Co., NY, USA) and stored at −80°C. Genomic DNA was extracted using a MasterPure DNA purification kit (Lucigen, WI, USA). DNA libraries were prepared using an Illumina DNA Prep kit (Illumina, San Diego, CA) and 150-bp paired-end reads were sequenced on a NextSeq 2000 at the Microbial Genome Sequencing Center (MiGS; Pittsburgh, PA). To see if the community response to MPn amendment was consistent across years, we performed further metagenomic sequencing on an MPn + N + C amendment from 2018 (July 24, dark incubation). The amendment was serially filtered through a 3-µm, 25-mm GTTP polycarbonate filter (EMD Millipore, MA, USA) onto a 25-mm, 0.2-µm polyethersulfone filter (SUPOR, Pall Co., NY, USA). Sample storage and DNA extraction were performed on the 0.2-µm filter as described above. Library preparation and shotgun 150-bp paired-end sequencing (150 bp) were performed on a Novaseq (Novogene, Sacramento, CA).

Read clean up, assembly, and functional annotation were performed as described for the *in situ* communities. To evaluate microbial community composition, 16S ribosomal RNA genes were identified using barrnap (100) and classified using the SINA Aligner (101) against the SILVA database (102). Only genes identified as prokaryotic were retained. The depth of coverage of each 16S rRNA gene was used to estimate the percent abundance of each organism within the community. We further compared metagenomes against one another based on functional potential. The coverage of genes with the same KEGG annotation was summed within each metagenome. KEGG abundances within each metagenome were rarefied to equal sampling depth and compared using NMDS ordinations based on Bray-Curtis dissimilarity. Permutational analysis of variance, based on the type of phosphorus added (MPn or phosphate), was performed using adonis in the package vegan (103). To explore the response of eukaryotic organisms, contigs predicted to belong to eukaryotes were identified using EukRep (104). The percentage of sequencing depth attributable to eukaryotic organisms in each metagenome was estimated based on the total reads that mapped to these contigs relative to the total number of reads.

Metagenome-assembled genomes were obtained using MetaBAT 2 v2.11.1 (105). Contigs >5 kb in length were retained. The size and quality of each genome bin were evaluated using QUAST v5.0.2 (106) and CheckM v1.0.13 (107). We report genome bins more than 60% complete and less than 10% contaminated, representing medium quality draft genomes (108). Genomes were taxonomically classified with GTDB-tk v1.6.0 (109) using KBase (110). Genome annotation was performed using Prokka v1.14.6 (111) and GhostKoala. Whole-genome trees of members of the genus *Acidovorax* were created using concatenated single-copy marker genes identified using CheckM, constructed using FastTree, and visualized using iTOL. Outgroups for Fig. 5B were members of the genus *Hydrogenophaga*. ANI comparisons were performed using OrthoANI (112). The relative representation of each bin within each metagenome was estimated by the number of reads mapped per kilobase million reads.

### 16S rRNA gene amplicon sequencing

To further evaluate the distribution of *Acidovorax* in Flathead Lake, we performed 16S rRNA gene amplicon sequencing at MLD in 2018. Water collection, DNA extraction, and sequencing were performed as previously described (113). Sequence data were processed using the QIIME2 platform (114). ASVs were identified using DADA2 (115) and classified against the SILVA 138 database (116). Further processing and visualization were performed using phyloseq (117) in R (118). Sequences related to chloroplasts and eukaryotes were removed. We used blastn to identify ASVs identical to the most abundant 16S rRNA gene related to *Acidovorax* in the 2020 MPn + N + C amendment. We also used this data set to identify potential methanogenic archaea and methanotrophic organisms, including the class Methylococcaceae.

## Data Availability

All sequence data are publicly available at NCBI under BioProject accession number PRJNA948362.

## SUPPLEMENTAL MATERIAL

Supplemental file 1 aem.01097-23-s0001.docx Fig. S1 to S8

Supplemental file 2 aem.01097-23-s0002.xlsx Table S1

ASM does not own the copyrights to Supplemental Material that may be linked to, or accessed through, an article. The authors have granted ASM a non-exclusive, world-wide license to publish the Supplemental Material files. Please contact the corresponding author directly for reuse.
