# Supplementary material for: Oxic methane production from methylphosphonate in a large oligotrophic lake: limitation by substrate and organic carbon supply
Source: Appl Environ Microbiol. 2023 Nov 30;89(12):e01097-23. doi: 10.1128/aem.01097-23 (PMC10734540; doi:10.1128/aem.01097-23)
Supplement: Supplemental file 1 — Fig. S1 to S8 [file aem.01097-23-s0001.docx]

**Supplemental Figure 1**. Relative abundance of the methanotrophic family Methylococcaceae in Flathead Lake based on 16S rRNA gene amplicon sequencing.


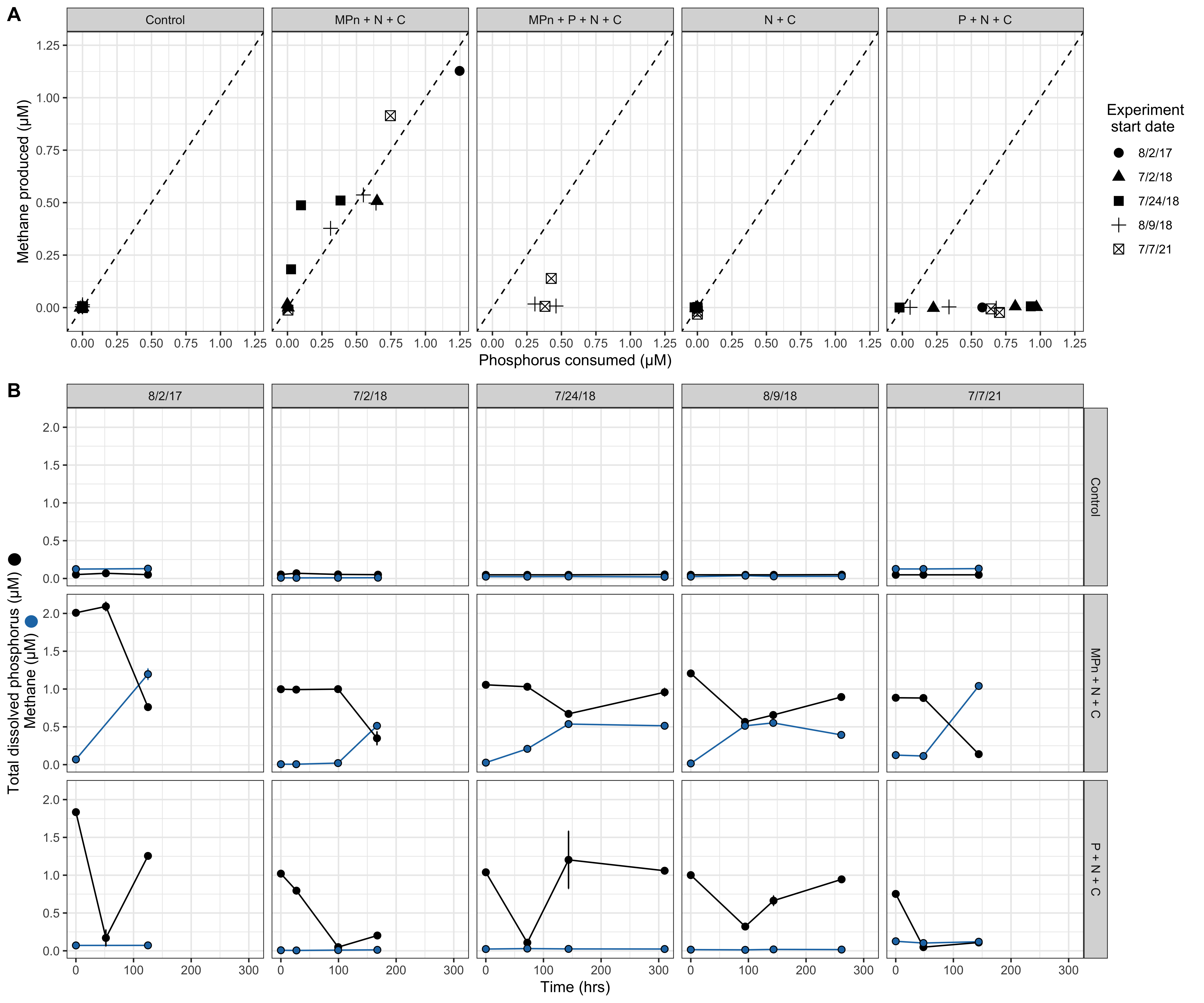


**Supplemental Figure 2**. Methane production is correlated with the consumption of methylphosphonate following nutrient amendment. A) Methane production compared to phosphorus (as total dissolved phosphorus) consumption during five experiments, with all methane and phosphorus measurements during the week-long experiments plotted. The dashed line represents the 1:1 line. Averages of replicates are shown. B) Total dissolved phosphorus (black) and methane (blue) concentrations over time in each experiment. The July 24, 2018 experiment was conducted in the dark.

**Supplemental Figure 3**. *In situ* conditions during sampling for the summer 2020 amendments, including methane and O_2_ saturation (left), temperature and photosynthetically active radiation (middle), and cell abundances (right; blue symbols reflect total picoplankton abundances, while black symbols reflect chlorophyll *a* containing cells).

**Supplemental Figure 4**. Nitrogen and phosphorus concentrations during the summer 2020 amendment showing that methylphosphonate appears less bioavailable than phosphate. A+C) NOx (nitrate+nitrite) or TDP (total dissolved phosphorus) concentrations at the start and end of each amendment. B+D) The percent of NOx or TDP consumed within each amendment.

**Supplemental Figure 5**. Members of the Burkholderiales (here in the class Gammaproteobacteria based on SILVA; alternatively, class Betaproteobacteria) responded to methylphosphonate addition in 2020. A) Metagenome-assembled 16S rRNA gene sequences which make up more than 3% of each community following nutrient amendment are shown. B) NMDS ordination of metagenomes based on Bray-Curtis dissimilarity of abundances of genes with KEGG annotations. Permutational analysis of variance using adonis revealed that the type of phosphorus added (MPn or phosphate, where the MPn+P+N+C treatment was designated as phosphate) was not a significant driver of functional potential (Pr(>F) = 0.1888).

**Supplemental Figure 6**. Photosynthetic organisms responded to amendment with P+N in 2020. Total cell abundances (A), photosynthetic cell abundances (B), and chlorophyll *a* concentration (C) at the beginning (T_0_) and end of the amendments. D) The percent of total reads in each amendment metagenome that mapped to predicted eukaryotic contigs.

**Supplemental Figure 7**. Metagenome-assembled genomes obtained represent the most abundant organisms in each amendment. Depth of coverage of contigs from metagenomes from five different amendments, as labeled across the top, including four from 2020 (A,B,C,D) and one from 2018 (E). Contigs longer than 10 kb are shown. Contigs in red represent binned MAGs of interest, including (A) *Fonsibacter* bin D13.bin.21, (B) *Flectobacillus* bin D16.bin.8, (C) *Acidovorax* bin D18.bin.11, (D) *Acidovorax* bin D19.bin.16, and (E) *Acidovorax* bin B02.bin.37. F) Read recruitment of genome bins belonging to the *Acidovorax* and *Rhodoferax* against different 2020 enrichment metagenomes indicate the presence of members of the same genera which respond in different ways to each amendment.

**Supplemental Figure 8**. Read recruitment of MAGs obtained from amendments against *in situ* metagenomes from Flathead Lake. MAGs which responded to MPn, such as *Acidovorax*, *Rhodoferax*, and *Allorhizobium*, appear rare *in situ*. *Fonsibacter*, which can represent up to 10% of Flathead Lake communities, is used here for comparison.
